# Combined Pulmonary Fibrosis and Emphysema: Pulmonary Function Testing and a Pathophysiology Perspective

**DOI:** 10.3390/medicina55090580

**Published:** 2019-09-10

**Authors:** Diana E. Amariei, Neal Dodia, Janaki Deepak, Stella E. Hines, Jeffrey R. Galvin, Sergei P. Atamas, Nevins W. Todd

**Affiliations:** 1 Department of Medicine, University of Maryland School of Medicine, Baltimore, MD 21201, USA; 2 Baltimore Veterans Affairs Medical Center, Baltimore, MD 21201, USA; 3 Department of Radiology and Nuclear Medicine, University of Maryland School of Medicine, Baltimore, MD 21201, USA

**Keywords:** emphysema, pulmonary fibrosis, elastic recoil, lung compliance, spirometry, lung volumes, diffusing capacity for carbon monoxide

## Abstract

Combined pulmonary fibrosis and emphysema (CPFE) has been increasingly recognized over the past 10–15 years as a clinical entity characterized by rather severe imaging and gas exchange abnormalities, but often only mild impairment in spirometric and lung volume indices. In this review, we explore the gas exchange and mechanical pathophysiologic abnormalities of pulmonary emphysema, pulmonary fibrosis, and combined emphysema and fibrosis with the goal of understanding how individual pathophysiologic observations in emphysema and fibrosis alone may impact clinical observations on pulmonary function testing (PFT) patterns in patients with CPFE. Lung elastance and lung compliance in patients with CPFE are likely intermediate between those of patients with emphysema and fibrosis alone, suggesting a counter-balancing effect of each individual process. The outcome of combined emphysema and fibrosis results in higher lung volumes overall on PFTs compared to patients with pulmonary fibrosis alone, and the forced expiratory volume in one second (FEV_1_)/forced vital capacity (FVC) ratio in CPFE patients is generally preserved despite the presence of emphysema on chest computed tomography (CT) imaging. Conversely, there appears to be an additive deleterious effect on gas exchange properties of the lungs, reflecting a loss of normally functioning alveolar capillary units and effective surface area available for gas exchange, and manifested by a uniformly observed severe reduction in the diffusing capacity for carbon monoxide (D_L_CO). Despite normal or only mildly impaired spirometric and lung volume indices, patients with CPFE are often severely functionally impaired with an overall rather poor prognosis. As chest CT imaging continues to be a frequent imaging modality in patients with cardiopulmonary disease, we expect that patients with a combination of pulmonary emphysema and pulmonary fibrosis will continue to be observed. Understanding the pathophysiology of this combined process and the abnormalities that manifest on PFT testing will likely be helpful to clinicians involved with the care of patients with CPFE.

## 1. Introduction

Pulmonary emphysema and pulmonary fibrosis are common chronic lung diseases which may result in mild pulmonary impairment or end-stage lung disease with chronic respiratory failure. Emphysema is most often caused by long-term exposure to cigarette smoke and is one of the major pathobiologic processes leading to the clinical phenotype of chronic obstructive pulmonary disease (COPD). Pulmonary fibrosis results from numerous diverse environmental, occupational or autoimmune etiologies and is included within the broad category of interstitial lung disease (ILD). Consequently, emphysema and fibrosis are often considered distinct entities with unique pathophysiologic manifestations, but over the past 10–15 years, there has been increasing recognition that these two processes may coexist in individual patients, and this overlapping disorder has often been termed combined emphysema and fibrosis or combined pulmonary fibrosis and emphysema (CPFE). During this time, there have been numerous original research studies, review articles and commentaries discussing the phenotype and physiologic characteristics of patients with CPFE, as well as the prognosis and clinical outcomes in these patients [1,2,3]. 

A common observation in patients with combined emphysema and fibrosis has been rather severe gas exchange abnormalities, manifested by hypoxemia and an almost universal moderate to severe reduction in diffusing capacity for carbon monoxide (DLCO) [1,2,3,4]. Despite these gas exchange abnormalities, one of the interesting and consistent findings across many studies has been only mild impairment in spirometric and lung volume indices, along with a normal forced expiratory volume in one second (FEV_1_)/forced vital capacity (FVC) ratio despite the presence of emphysema on chest CT imaging [1,2,3,4]. The presence of only mild impairment in spirometry along with a preserved FEV_1_/FVC ratio has led to the consideration that the abnormal respiratory mechanics of emphysema are being counterbalanced in some manner by the abnormal respiratory mechanics of pulmonary fibrosis. This observation has been sometimes referred to as pseudo-normalization of spirometry and lung volume parameters in these patients with CPFE [5,6]. 

The purpose of this review is to discuss pathophysiologic disturbances in pulmonary emphysema, pulmonary fibrosis, and combined emphysema and fibrosis, and to discuss the results of numerous studies which have described pulmonary function (PFT) parameters in these CPFE patients. Hopefully this discussion will help the reader better understand PFT findings and gas exchange abnormalities in patients with combined emphysema and fibrosis. 

## 2. Pulmonary Emphysema

Emphysema is a chronic lung disease characterized pathologically by destruction of extracellular matrix and enlargement of airspaces distal to the terminal bronchiole [7]. Emphysema is most commonly caused by exposure to cigarette smoke, although may be caused by exposure to a wide array of environmental vapors, fumes or dusts, and is most commonly identified by low attenuation areas of the lung parenchyma without clearly definable walls on computed tomography (CT) scan of the chest [8,9,10].

The presence of emphysema has several pathophysiologic consequences which cause both gas exchange and mechanical abnormalities. Within emphysematous spaces, ventilation is impaired, but perfusion is generally impaired to a greater degree or may be completely absent, leading to the widespread development of alveolar capillary units with high ventilation-perfusion ratios [11,12]. Alveolar capillary units with high ventilation-perfusion ratios lead to increased physiologic dead space, inefficient (or wasted) ventilation, and increased minute ventilation requirements which are necessary to preserve alveolar ventilation. Worsening hyperinflation of the lungs during an exacerbation in patients with emphysema may additionally contribute to further ventilation-perfusion imbalance [13]. An inability over time to maintain a normal level of alveolar ventilation will lead to both chronic hypoxemia and hypercapnia [14,15]. High minute ventilation requirements in emphysema result in increased work of breathing, increased overall energy expenditure, and at times cachexia in patients with advanced emphysema [16]. Due to the destruction of extracellular matrix in emphysema, the number of normally functioning alveolar capillary units is reduced, resulting in a loss of effective surface area for gas exchange. Anatomic loss of effective surface area will be manifested by a reduced diffusing capacity of the lung for carbon monoxide (D_L_CO) and will be associated physiologically with ventilation-perfusion inequality, which in emphysema will be mostly areas of high ventilation-perfusion mismatch. A reduced D_L_CO in emphysema has been correlated with overall severity of disease and oxygen desaturation with exercise [17,18]. 

In regard to lung mechanics, destruction of extracellular matrix and enlargement of air spaces in emphysema causes a reduction in elastic recoil forces of the lung [11,19]. Due to the inverse relationship between elastance and compliance, lung compliance (distensibility of the lung) in emphysema is increased. A reduction in elastic recoil forces of the lung in emphysema results in several unwanted outcomes. First, airway collapse on forced expiration is accentuated, resulting in a reduced FEV_1_ and reduced FEV_1_/FVC ratio, and thus an obstructive ventilatory defect on spirometry. Along with intrinsic airway resistance, lung elastic recoil forces are a major determinant of the amount of airflow on expiration. Elastic recoil forces provide radial traction support to small airways during the breathing cycle, in addition to their effect on lung compliance. When elastic recoil forces are reduced, radial traction forces on small airways are reduced, and on subsequent forced expiration, airway collapse is accentuated and FEV_1_, FEV_1_/FVC, and rates of airflow are all reduced. All normal healthy persons have some degree of airway collapse on forced expiration, manifested on spirometry as a FEV_1_/FVC ratio of approximately 0.75–0.80 in healthy middle-aged adults, but in patients with emphysema, airway collapse on forced expiration is excessive, and the expiratory time constant, a product of lung compliance and airway resistance, is prolonged [20].

Second, a reduction in elastic recoil forces results in an increased functional residual capacity (FRC) and total lung capacity (TLC), often termed static hyperinflation [11,21]. Resting FRC (or end-expiratory lung volume (EELV)) is defined as the lung volume at which elastic recoil forces of the lung inward are numerically equal but opposite to elastic recoil forces of the chest wall outward [14,21]. Since elastic recoil forces in emphysema are reduced, the resting volume at which inward lung forces equilibrate with outward chest wall forces occurs at a higher lung volume. Similarly, TLC is often increased in emphysema due to reduced inward elastic recoil forces of the lung at full inspiration, as assessed by pressure-volume curves in emphysema patients. [11,21]. Since TLC is usually only mildly increased, which is due to normal chest wall mechanics and compliance in emphysema limiting the extent to which the thorax can ultimately expand, an elevated FRC reduces inspiratory capacity (IC) and limits the increase in tidal volume (V_T_) which occurs in response to increased ventilatory demands from exercise or a variety of other stressors. Additionally, in emphysema, EELV may increase even further during exercise (termed dynamic hyperinflation), which occurs due to expiratory airflow limitation coupled with shortened expiratory time, and which further reduces IC, alters lung compliance, and limits potential increases in V_T_ [21]. Dynamic hyperinflation can additionally impair venous return to the thorax, further impairing gas exchange and cardiopulmonary hemodynamics.

Third, residual volume (RV) is increased in emphysema, often termed air trapping. Residual volume in the normal lung results from closure of airways at the end of exhalation, and is elevated in emphysema since reduced elastic recoil forces lead to airway closure at a lung volume which is abnormally high [19]. Increased propensity to airway closure in emphysema is also reflected in an abnormally high closing volume (CV) and closing capacity (CC), both of which can be determined from the single breath nitrogen washout test and refer to the lung volume above RV at which the onset of airway closure occurs [22,23]. A substantially elevated RV in the presence of a mildly increased TLC leads to an elevated RV/TLC ratio in emphysema, and results in a significantly reduced vital capacity (VC). Overall, in addition to gas exchange abnormalities which are present, reduced elastic recoil forces in emphysema result in static hyperinflation, dynamic hyperinflation, and excessive airflow limitation on expiration, all of which are likely major contributors to resting dyspnea and reduced exercise capacity in patients with emphysema [21,24].

## 3. Pulmonary Fibrosis

In contrast to emphysema, pulmonary fibrosis has most often been described as a pathobiologic process in which an excess of extracellular matrix accumulates, and is characterized by a combination of excessive extracellular matrix production, alveolar epithelial cell loss, and permanent alveolar collapse [25,26,27,28]. The effects of pulmonary fibrosis on gas exchange and mechanical properties of the lung are well characterized. Pulmonary fibrosis may be caused by a myriad of environmental exposures, medication toxicities, or autoimmune mechanisms, or may develop without a well-defined etiology and be identified as idiopathic pulmonary fibrosis (IPF) [29]. Pulmonary fibrosis is identified on chest CT imaging by a combination of findings including reticulation, volume loss, traction bronchiectasis, and honeycombing [30].

The development of pulmonary fibrosis alters ventilation-perfusion relationships within the lung [12,15,19]. In the fibrotic lung, there are likely numerous areas in which alveolar capillary units demonstrate low ventilation-perfusion ratios, characterized by greater impairment in ventilation compared to perfusion at the alveolar level. Alveolar capillary units with low ventilation-perfusion ratios and those with shunt (absent ventilation with preserved perfusion) cause hypoxemia both at rest and with exertion, and patients with advanced pulmonary fibrosis almost always require supplemental oxygen. Although ventilation-perfusion mismatch is likely the major cause of hypoxemia in patients with pulmonary fibrosis, a diffusion impairment in oxygen transfer may additionally contribute to hypoxemia both at rest and on exertion in patients with severe disease as a result of increased thickness of the alveolar capillary membrane [14,15,31]. In other areas of the fibrotic lung, there are likely alveolar capillary units with high ventilation-perfusion ratios, and along with areas of true dead space (absent perfusion with preserved ventilation), result in inefficient ventilation and higher minute ventilation requirements in order to preserve alveolar ventilation. Similar to emphysema but albeit by different mechanisms, the number of normally functioning alveolar capillary units in pulmonary fibrosis is reduced, likely resulting from a combination of excess production of extracellular matrix, pulmonary vascular abnormalities, alveolar epithelial cell loss and permanent alveolar collapse. This loss of normally functioning alveolar capillary units results in a loss of effective surface area for gas exchange, ventilation-perfusion inequality, and a reduced D_L_CO [12,32,33]. A reduced D_L_CO is associated with severity of disease and poor outcomes in patients with pulmonary fibrosis [34,35].

In contrast to emphysema, the development of pulmonary fibrosis results in increased elastic recoil forces of the lung (thus increased elastance) and therefore reduced lung compliance (reduced distensibility of the lung) [19,31]. The increased elastance and reduced compliance result in overall low lung volumes in most patients, manifested as a reduction in FEV_1_, VC, FRC, and TLC [19,31,36], and thus full pulmonary function testing (PFTs) will manifest a restrictive ventilatory defect. Resting FRC is reduced since the increased inward recoil force of the lung will equilibrate with the outward recoil force of the chest wall at a lower lung volume, and TLC is mildly to moderately reduced due to markedly increased inward elastic recoil forces of the lung at full inspiration [36]. RV is often relatively preserved in pulmonary fibrosis, especially if cystic change (honeycombing) and/or small airway disease are present as part of the fibrotic process [36]. Since TLC is usually decreased proportionally to a greater degree than RV, the RV/TLC ratio is often elevated and the VC is usually significantly reduced [33,36,37]. Lung volumes including VC and TLC may be relatively preserved in early or mild fibrotic disease due to normal chest wall compliance and normal respiratory muscle strength, even though lung elastance is uniformly increased. The FEV_1_/FVC ratio in pulmonary fibrosis is preserved or even increased above normal in most instances, even though the absolute values of FEV_1_ and FVC are reduced [32,33,36]. This occurs as a result of increased elastic recoil forces in pulmonary fibrosis which tether small airways open and therefore reduce the tendency for airway collapse on forced expiration. A significantly elevated FEV_1_/FVC ratio should alert the clinician to the possibility of ILD and/or pulmonary fibrosis. 

Work of breathing is also elevated in patients with pulmonary fibrosis during normal breathing and with exercise as a result of the work required to overcome the reduced compliance and increased elastance [36,38]. The constellation of gas exchange abnormalities, increased lung elastance, reduced ventilatory capacity and increased work of breathing all lead to dyspnea and reduced exercise capacity in patients with pulmonary fibrosis.

## 4. Combined Emphysema and Fibrosis

There has been increasing recognition over the past 10–15 years that pulmonary emphysema and pulmonary fibrosis may coexist in individual patients and has been most often referred to as CPFE. In the above sections, we have discussed the abnormalities in elastance and compliance that occur in isolated emphysema and isolated pulmonary fibrosis. From a simplistic view, one could postulate that the mechanical abnormalities resulting from a combination of emphysema and fibrosis would be additive in a deleterious way, resulting in a further decline in spirometric and lung volume indices compared to the presence of either process alone. This additive concept appears to be valid when applied to gas exchange properties of the lungs, as patients with CPFE have severe gas exchange abnormalities manifested by a severe reduction in D_L_CO (Table 1). This D_L_CO observation seems very plausible since both emphysema and fibrosis reduce the number of normally functioning alveolar capillary units and thus reduce the effective surface area available for gas exchange. In regard to mechanical properties of the lungs however, patients with CPFE have been shown to most often have only mild impairment in spirometric and lung volume indices despite rather severe imaging and gas exchange abnormalities. This observation suggests the abnormal lung mechanics of each individual process are likely being counter-balanced which results in relative preservation of lung volumes and elastic recoil properties [1,2,4,5,6,39]. 

The precise pathobiology which leads to CPFE in individual patients likely has various mechanisms, although cigarette smoke appears to have a major role. From a genetic standpoint, numerous studies have examined genetic profiling in patients with advanced emphysema, and abnormalities have been observed in mechanisms related to inflammation, oxidative stress, extracellular matrix synthesis, epithelial and endothelial cell function, cell-cell signaling, cell migration and cell senescence [40,41]. Recent genome-wide association studies have also identified numerous loci related to the function of endothelial cells, type I and II alveolar epithelial cells, fibroblasts and smooth muscle cells in COPD [42] and familial forms of emphysema have been caused by mutations related to telomere biology and protease-anti-protease imbalance (alpha-1 antitrypsin) [43]. In pulmonary fibrosis, numerous studies have likewise examined gene expression profiling, and abnormalities in mechanisms related to inflammation, immune system activation, intracellular metabolic processes, alveolar epithelial cells, mesenchymal cells and cell senescence have been observed [28,44,45]. Additionally, mutations have been identified in individual genes in patients with familial forms of pulmonary fibrosis, and have included mutations related to surfactant protein biology, telomere biology, and mucin biology [46,47]. Based on the above, there appear to be numerous overlapping mechanistic abnormalities in gene expression in patients with pulmonary emphysema and patients with pulmonary fibrosis [48,49]. To our knowledge, only a few studies have examined genetics related to CPFE. One recent study demonstrated enrichment of immunoglobulin associated genes in the fibrotic areas of CPFE, whereas enrichment of genes related to cell membrane structures and vascular growth was observed in the emphysematous areas [50]. Based on all current available evidence of genetic profiling in patients with these diseases, it is unknown at the present time why individual patients with a history of smoking develop pulmonary emphysema, pulmonary fibrosis, or CPFE.

Three potential timelines of disease development in CPFE could be postulated. Pulmonary emphysema may develop initially over many years as the initial injury process due to cigarette smoking and followed subsequently at some later time point by development of pulmonary fibrosis. The development of pulmonary fibrosis in this scenario may also be a result of long-term smoking, since cigarette smoke is a well-documented, but likely often overlooked, cause of excess collagen accumulation and fibrosis in the lung [51,52]. Alternatively, the development of fibrosis in this timeline may result from any number of environmental/avocational/occupational exposures or autoimmune mechanisms which are unrelated to both cigarette smoking and the presence of emphysema. Second, emphysema and fibrosis may both develop concurrently as a result of cigarette smoking, and in this instance, the findings of emphysema and fibrosis are likely to be located in a similar geographic distribution within the lung. Third, pulmonary fibrosis may be the initial process to develop which is then followed subsequently by the development of emphysema. This scenario may not seem quite as plausible based on clinical observations, but inflammation, fibrosis, and destruction of small airways as part of cigarette smoke-induced injury has been postulated as a precursor to development of parenchymal emphysema in some instances [53,54]. Additionally, centrilobular emphysema is generally considered the prototype of cigarette smoking-induced emphysema, but many patients with pulmonary fibrosis exhibit significant paraseptal emphysema, suggesting that the fibrotic process in some manner may be contributing to the development of paraseptal emphysema [55,56,57]. 

**Figure 1:**
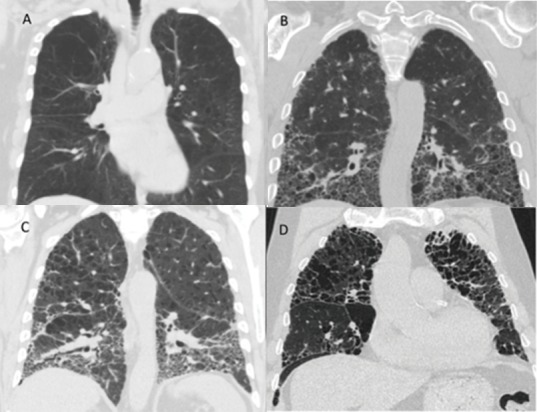
Representative chest computed tomography (CT) images from selected patients from the authors’ institution with emphysema alone (**A**), pulmonary fibrosis alone (**B**), upper lobe emphysema with lower lobe pulmonary fibrosis (**C**), and upper lobe emphysema with thickened fibrotic walls, also referred to as air space enlargement with fibrosis (**D**).

Various CT imaging patterns have been observed in patients with combined emphysema and fibrosis (Figure 1). Patients may demonstrate predominantly emphysema in the upper lobes and predominantly pulmonary fibrosis in the lower lobes (Figure 1C), emphysema in the upper lobes with diffuse ground-glass opacities and fibrotic change both in the upper and lower lobes, or enlarged emphysematous spaces surrounded by thickened fibrotic walls often in a predominantly upper lobe distribution (Figure 1D). In this latter pattern, the findings of emphysema and fibrosis exist in a similar geographic distribution within the lung, and this pattern has often been referred to as airspace enlargement with fibrosis [58,59]. Each of these different radiographic patterns can be described under the broad heading of CPFE, which highlights the lack of a universal precise characterization of patients with CPFE. One could speculate on whether there is a correlation between a particular imaging pattern and a particular timeline of disease development as described in the preceding paragraph, but an accurate association in this regard is unknown at present.

Elastic recoil properties of the lungs and lung compliance are difficult to measure routinely in the clinical setting, but the effects of these processes can be assessed with standard spirometry and lung volume testing. In the ensuing sections, we will discuss the results of published studies which have compared standard PFT indices in patients with CPFE to those with pulmonary fibrosis alone. Despite differing imaging patterns of combined emphysema and fibrosis, it seems plausible that the resulting effects on lung mechanics manifested on standard PFT testing would be similar, although patients with basilar fibrosis in CPFE may be expected to have more gas exchange impairment due to greater lower lobe perfusion in general. 

Table 1 shows the results of 11 studies over the past 15 years in which PFT parameters in patients with pulmonary fibrosis alone (PF) were compared to patients with CPFE, and each study had at least ten patients in the CPFE group. The primary objectives of each of these studies were not always identical, as some were aimed at outcomes in these two patient groups, some at the value of a composite physiologic index (CPI) for patients with fibrosis and emphysema, and some at longitudinal PFT changes in CPFE patients, but this published data does allow a reasonable comparison of PFT parameters from these two patient groups. Most studies that have examined PFTs in CPFE have compared CPFE to PF, but one recent study did compare patients with CPFE to patients with COPD [57] and extending the comparison of pulmonary function in CPFE to emphysema alone seems reasonable given the numerous published PFT studies in patients with emphysema over many years [60,61].

## 5. Total Lung Capacity (TLC)

As seen in Table 1, TLC was higher in patients with CPFE compared to patients with PF alone, and TLC was either normal or close to normal in CPFE patients in most of the studies. TLC is elevated in most patients with emphysema [61] and mildly to moderately reduced in most patients with PF alone, as seen in Table 1, and thus reduced elastic recoil forces associated with emphysema are likely resulting in a higher TLC in CPFE patients compared to patients with PF alone. One other study demonstrated that in patients with IPF, a greater number of pack-years of smoking correlated with an increased TLC, although this study did not directly compare patients with PF alone to CPFE [62]. Overall, most published data support the concept that patients with CPFE usually have an intermediate value for TLC which rests between higher values associated with emphysema alone and lower values associated with fibrosis alone.

## 6. Functional Residual Capacity (FRC)

Only one study in Table 1 published values for FRC, and thus we did not include FRC results in the Table. In this study, FRC percent predicted was 76 ± 22 in PF compared to 78 ± 19 in CPFE, which was not statistically different [63]. In our previous study which is shown in Table 1, we did not originally publish our FRC data, but values recorded for FRC percent predicted were 54 ± 19 in PF compared to 75 ± 32 in CPFE (p = 0.0014) [55]. Similar to TLC, a prior study demonstrated that a greater number of pack-years of smoking in patients with IPF correlated with an increased FRC, although again this study did not directly compare patients with PF alone to CPFE [62]. Overall, based on principles of elastic recoil, it would seem plausible that FRC values in patients with CPFE would be higher than patients with PF alone, but there is not much published data in this regard.

## 7. Residual Volume (RV)

Six of the studies in Table 1 reported values for RV. In three of the studies, RV was significantly higher in patients with CPFE compared to PF alone, whereas RV was statistically unchanged in the remaining three. Similar to FRC and TLC, a greater number of pack-years of smoking correlated with an increased RV in patients with IPF [62] Since patients with advanced emphysema will uniformly have an elevated RV [60,61], it seems possible that the reduced elastic recoil forces related to the emphysematous component in CPFE would lead to lead to a higher RV in these patients compared to PF alone. However, RV may be relatively preserved even in patients with advanced pulmonary fibrosis [36], which may lead to not much overall difference in RV when comparing CPFE patients to those with PF alone. Overall, there is not a lot of published data on RV in patients with CPFE.

## 8. Forced Vital Capacity (FVC)

As seen in Table 1, FVC was uniformly higher in patients with CPFE compared to those with PF alone, having reached statistical significance in seven of the 11 studies and trended higher in the remaining four. Since FVC is the difference in volume between TLC and RV, patients with CPFE must in some combination have a greater volume difference between TLC and RV. As seen in Table 1, the majority of studies demonstrate higher TLC values in patients with CPFE compared to PF alone, whereas RV results have been either variable or not reported. More published data on RV values in patients with CPFE would likely help clarify conclusions in regard to relationships between FVC, TLC and RV in these patients. 

## 9. Forced Expiratory Volume in One Second (FEV_1_)/Forced Vital Capacity (FVC) Ratio

Although the FEV_1_/FVC ratio was generally preserved in CPFE patients and above 0.70 in most studies, the ratio was rather uniformly reduced in patients with CPFE compared to PF alone, having reached statistical significance in nine of the 11 studies and trended lower in the remaining two, as seen in Table 1. The lower FEV_1_/FVC ratio in patients with CPFE compared to patients with PF alone suggests differing elastic recoil forces in these two groups of patients, which may potentially be related to the emphysema component in patients with CPFE.

## 10. Forced Expiratory Volume in One Second (FEV_1_)

As shown in Table 1, eight studies reported FEV_1_ and all but one of the studies showed no statistically significant difference in the FEV_1_ between the CPFE and PF groups. This may be a little counterintuitive if all lung volumes in general are higher in CPFE patients, but is consistent with the data for FVC and FEV_1_/FVC ratio described above. Even though FVC is higher on average in patients with CPFE compared to PF, the FEV_1_/FVC ratio is lower on average, and thus having an FEV_1_ value which is not different between the two groups seems very plausible. Overall, FEV_1_ values expressed as percent predicted appear normal or mildly reduced in most studies of CPFE.

## 11. Diffusing Capacity for Carbon Monoxide (DLCO)

The D_L_CO is moderate to severely low in patients with CPFE, as shown in Table 1, which is a very consistent and characteristic finding in all studies of CPFE patients [2], and is consistent with the effects of each individual process (emphysema and fibrosis) reducing the amount of effective surface area that is available for gas exchange. In five of the studies, a statistically lower D_L_CO value was observed in the CPFE compared to the PF patients, consistent with an additive effect of emphysema combined with fibrosis, whereas D_L_CO was not different between the two groups in the remaining studies.

Although not specific to CPFE, one concept which deserves mentioning in regard to assessment of D_L_CO is the D_L_CO/V_A_ [31,64,65,66], as the D_L_CO/V_A_ has been reported in some studies of CPFE patients in addition to reporting D_L_CO. The D_L_CO is calculated by multiplying the alveolar volume (V_A_) by the K_CO_, which is the rate constant for uptake of CO from alveolar gas. Therefore, K_CO_ can be and is often expressed as D_L_CO/V_A_. Evaluating and drawing conclusions from D_L_CO/V_A_ has been discussed for many years in pulmonary medicine, likely with the goal of attempting to assess whether a reduced D_L_CO is merely a reflection of reduced lung volume or is in fact reduced as a result of abnormal gas exchange properties of the lung in addition to any lung volume reduction. As many authors have pointed out, interpreting the D_L_CO/V_A_ can be complex. It has been well-established that D_L_CO/V_A_ (K_CO_) is not constant as lung volume changes, should not be interpreted as a correction for lung volume, and actually increases as lung volume decreases from TLC to FRC in the healthy adult [31,64,65]. With standard PFT testing, predicted values for D_L_CO/V_A_ are provided for a normal TLC volume, thus there are no routinely provided predicted values for D_L_CO/V_A_ at differing lung volumes in individual patients. Consequently, in our judgment, assessing the measured D_L_CO as opposed to the D_L_CO/V_A_ is likely to provide a more accurate assessment of the true gas exchange properties of the lungs in patients with CPFE. 

**Table 1:** Published pulmonary function test (PFT) parameters comparing patients with pulmonary fibrosis alone (PF) to patients with combined pulmonary fibrosis and emphysema (CPFE).

Year	FVC % Predicted ± SD		FEV_1_ % Predicted ± SD		FEV_1_/FVC Ratio ± SD		TLC % Predicted ± SD		RV % Predicted ± SD		DLCO % Predicted ± SD		Fibrosis Score
	*PF*	*CPFE*	*PF*	*CPFE*	*PF*	*CPFE*	*PF*	*CPFE*	*PF*	*CPFE*	*PF*	*CPFE*	
2006 [67]	70 ± 22	77 ± 20	77 ± 26	76 ± 31	0.83 ± 0.07	0.74 ± 0.18	71 ± 18	95 ± 25 *	73 ± 29	111 ± 49 *	49 ± 18	48 ± 26	similar
2009 [56]	59 ± 18	62 ± 16	67 ± 20	70 ± 15	0.93 ± 0.11	0.91 ± 0.09	nr	nr	nr	nr	nr	nr	*
2009 [63]	73 ± 19	86 ± 24 *	nr	nr	0.85 ± 0.07	0.77 ± 0.09 *	70 ± 16	78 ± 17	76 ± 27	75 ± 23	61 ± 20	45 ± 15 *	nr
2010 [68]	62 ± 16	77 ± 14 *	67 ± 15	71 ± 20	0.78 ± 0.09	0.67 ± 0.12 *	66 ± 15	76 ± 11 *	73 ± 30	75 ± 24	50 ± 22	29 ± 11 *	nr
2010 [69]	72 ± 19	87 ± 17 *	87 ± 21	88 ± 20	0.81 ± 0.08	0.70 ± 0.12 *	77 ± 16	94 ± 17 *	nr	nr	74 ± 20	65 ± 21 *	similar
2011 [70]	65 ± 14	76 ± 15	77 ± 17	84 ± 16	0.84 ± 0.06	0.78 ± 0.07 *	nr	nr	nr	nr	46 ± 14	42 ± 16	nr
2011 [55] ^§^	51 ± 17	64 ± 19 *	59 ± 20	66 ± 15	0.84 ± 0.06	0.78 ± 0.10 *	50 ± 16	66 ± 16 *	49 ± 19	77 ± 35 *^,†^	30 ± 15	29 ± 14	similar
2013 [1]	65 ± 17	80 ± 16 *	71 ± 18	80 ± 17 *	0.83 ± 0.07	0.74 ± 0.06 *	66 ± 13	79 ± 14 *	66 ± 23	74 ± 30	44 ± 15	37 ± 14 *	*
2014 [71]	74 ± 19	94 ± 22 *	nr	nr	0.86 ± 0.07	0.78 ± 0.12 *	72 ± 15	90 ± 16 *	78 ± 22	99 ± 27 *	54 ± 18	50 ± 14	*
2014 [72]	68 ± 28	83 ± 22	nr	nr	0.82 ± 0.04	0.77 ± 0.03 *	nr	nr	nr	nr	57 ± 27	37 ± 18 *	nr
2019 [73]	75 ± 16	93 ± 12 *	88 ± 18	96 ± 14	0.83 ± 0.08	0.72 ± 0.10 *	74 ± 13	90 ± 11 *	nr	nr	67± 19	58 ± 21	similar

FVC = Forced vital capacity; FEV1 = Forced expiratory volume in one second; TLC = Total lung capacity; RV = Residual volume; DLCO = Diffusing capacity for carbon monoxide; SD = Standard deviation; PF = Pulmonary fibrosis alone; CPFE = Combined pulmonary fibrosis and emphysema; * indicates significant difference (*p* < 0.05); nr = not reported; ^§^ data originally published as median with quartiles, but recalculated and presented here as mean ± SD; ^†^ residual volume data collected as part of initial study, but not reported with prior publication.

## 12. Overall Observations from Table 1

The published studies in Table 1 provide an overview of PFT parameters in patients with CPFE compared to PF alone, but one potential limitation in drawing conclusions from these studies relates to assessing the degree of parenchymal fibrosis in each group. Fibrosis scoring is generally performed utilizing a scoring system based on chest CT imaging, although scoring fibrosis in the presence of emphysema may be difficult [1]. If the degree of parenchymal fibrosis in PF patients is higher compared to CPFE patients, the higher lung volumes observed in CPFE patients may merely result from less fibrosis as opposed to changes in elastic recoil forces due to the emphysema component. As seen in the far right column in Table 1, three studies did show a statistical difference in fibrosis scores between the two groups of patients, whereas there was no difference or were not reported in the remaining studies. Additionally, some of the conflicting results among the published studies may result from patients having different geographic forms of emphysema and fibrosis, again highlighting that there is no universal precise characterization of patients with CPFE. Despite these limitations, overall observations in aggregate from the studies shown in Table 1 and other studies of patients with CPFE have demonstrated that the abnormal lung mechanics of each individual process, emphysema and fibrosis, likely cause a counter-balancing effect and result in relative preservation of spirometric and lung volume indices. 

## 13. Conclusions

In this Review, we have examined the physiologic characteristics that occur with pulmonary emphysema, pulmonary fibrosis, and CPFE. As we have hopefully demonstrated, the preponderance of literature suggests that patients with CPFE have relative preservation of spirometric and lung volume indices, but have severe gas exchange abnormalities [2,3,4]. From a physiologic standpoint, these observations suggest that elastic recoil properties of the lungs in patients with CPFE are likely intermediate between those of patients with pulmonary fibrosis and those with emphysema, suggesting a counter-balancing effect. As emphasized in many prior publications, patients with CPFE, who may be severely functionally impaired with advanced illness and a poor prognosis, may have normal or only mildly impaired spirometry and lung volume indices, which may provide a false sense of normalcy in a patient with substantially abnormal pulmonary physiology. In all studies of CPFE patients, D_L_CO has been uniformly profoundly reduced, suggesting that D_L_CO does likely reflect the severity of illness in these patients and emphasizes the importance of the D_L_CO measurement when the diagnosis of CPFE is considered. As chest CT imaging continues to be a frequent imaging modality in patients with cardiopulmonary disease, we expect that patients with a combination of pulmonary emphysema and pulmonary fibrosis will continue to be observed, and understanding the abnormalities that manifest on PFT testing can be helpful to understanding the overall pathophysiology of disease in these patients.
